# Type 2 diabetes mellitus Awareness in a Sample of South Asians in the United States

**DOI:** 10.1038/s41598-026-52175-6

**Published:** 2026-05-07

**Authors:** Emily Peter, Pallavi Sripathi, Ananta Srivastava, Fanglong Dong, Maryam Othman

**Affiliations:** 1 College of Osteopathic Medicine of the Pacific, Western University of Health Sciences, Pomona, CA USA; 2 College of Podiatric Medicine, Western University of Health Sciences, Pomona, CA USA

**Keywords:** Type 2 diabetes mellitus, South Asian, Education, United States, Diseases, Endocrinology, Health care, Medical research

## Abstract

South Asians living in the United States experience disproportionately higher rates of type 2 diabetes mellitus (T2DM) as compared to other groups. Prior research demonstrates that diabetes education improves health outcomes. This study assesses U.S. South Asian knowledge about T2DM. This cross-sectional study was conducted from August 1st to August 14th, 2024, and utilized two previously validated surveys. Eligibility criteria involved being a South Asian and a U.S. citizen or permanent resident. Participants were recruited via snowball sampling through social media platforms. The 22 survey questions were grouped into three categories: diabetes general knowledge (DK), diabetes management (DM), and complication management (CM). A total of 215 of the 219 survey respondents met inclusion criteria. 17 of the 22 survey items were answered correctly by more than 80% of participants. DK questions were answered with the lowest average accuracy (80.7%). Participants diagnosed with T2DM and healthcare workers answered questions with statistically higher accuracy in more than one question category in the univariate analysis (e.g., in the DK category: *p* = 0.0225, Cohen’s d = 0.42, and *p* = 0.0002, Cohen’s d = 0.60, respectively), and these findings remained largely consistent after adjusting for other variables in the multivariate analysis. The two most frequently missed questions were related to hypoglycemic symptoms. This study is limited by the use of convenience sampling, which may reduce generalizability to the broader U.S. South Asian population. Future research goals involve gathering a more representative sample of U.S. South Asians, as this study involved a predominantly Indian and highly educated subpopulation, and investigating the utility of community-based initiatives for T2DM education and promotion of healthy behaviors.

## Introduction

Type 2 diabetes mellitus (T2DM) is a chronic condition associated with increased risk for all-cause mortality^1^. The prevalence and incidence of T2DM among South Asians living in the United States exceeds that of their non-Hispanic White counterparts^2^. Recent studies estimate a T2DM disease prevalence of 27% in South Asians living in the United States as compared to 8% in non-Hispanic White Americans^2^. Additionally, the incidence of this condition is markedly elevated at 18.9 per 1000 person-years for South Asians in the United States compared to 11.1 per 1000 person-years for non-Hispanic White Americans^2^.

This heightened vulnerability may be driven by the unique metabolic profile of South Asians characterized as having a paradoxical relationship between increased body fat percentage and a relatively low body mass index (BMI)^3^. Notably, one study demonstrated that Indian Americans within the United States had an increased cardiovascular risk that was specifically driven by a risk factor of having low high-density lipoprotein (HDL)^3^. One of the significant correlates identified with low HDL included a lack of knowledge about diabetes, highlighting that health literacy can be a critical determinant of increased risk of T2DM within this population^3^. This is corroborated by findings of the MASALA study, where South Asians possess a greater volume of ectopic fat compared to other groups with a low BMI, and as such, using traditional criteria for T2DM screening with weight may lead to the under-identification of several individuals with this metabolic profile^2^. Furthermore, research delving into Metabolic Syndrome among Asian Indians in the United States concluded that even with high prevalence, with rates upwards of 52.5%, there was no corresponding increase in health promotion behaviors among those affected^4^. This lack of engagement is further complicated by cultural and systemic barriers regarding targeted dietary modification in South Asians with T2DM, as studies show that this intervention may fail because of cultural insensitivity and community misconceptions^5^. Collectively, these studies provide insight into the various components that may contribute to disproportionate rates of T2DM within South Asian communities in the United States.

Prior studies suggest that T2DM education confers improved glycemic control, and ultimately, better health outcomes in patients^6–8^. Research from the United Kingdom demonstrated that South Asians with T2DM had decreased awareness about their diagnosis as compared to other ethnic groups^9^. There are no similar studies assessing diabetes knowledge among South Asian communities living in the United States. This gap requires further exploration, as barriers such as poor health literacy in T2DM, can make clinical management significantly more difficult^10^. Given the increased vulnerability of this population to T2DM with documented behavioral gaps, it is crucial to assess baseline knowledge of the disease in this community as education could serve as a key intervention strategy. This preliminary cross-sectional study aims to evaluate the existing awareness about T2DM among South Asian communities living in the United States.

## Methods

### Setting and study population

Participants were eligible for this cross-sectional study if they were South Asian, 18 years or older, a U.S. Citizen or a permanent resident, and spoke English. An individual was considered South Asian if they had an ethnic background from one or more of the following countries: Afghanistan, Bangladesh, Bhutan, India, Maldives, Nepal, Pakistan, and Sri Lanka^11^. The survey was disseminated through direct messaging platforms, such as iMessage and WhatsApp, from August 1st through August 14th, 2024, using a snowball sampling method. Investigators sent out surveys to their personal contacts, who were subsequently asked to distribute it to others within their networks. As this was a preliminary study, data collection was planned to stop once either 100 participants were reached or three months of data collection had elapsed, whichever occurred first. The survey began with a section detailing the intent of the project as well as a disclosure that participation was voluntary and anonymous. This section also explained that submitting the survey would serve as informed consent for the results to be used in future presentations and publications. This study was approved by the Institutional Review Board at Western University of Health Sciences (approval number 2207366-3) and was performed in compliance with all guidelines.

### Survey instrument contents

The survey was created on Google Forms and consisted of 34 questions. Settings were configured in Google Forms such that the survey could not be submitted unless all questions were completed. As a result, the dataset contained no missing responses. There were 12 demographic and 22 diabetes knowledge assessment questions. The demographic information included age, sex, ethnicity, level of education, relationship to the healthcare system, and clinical diagnoses. The knowledge assessment questions were drawn from two previously validated surveys: “Diabetes Knowledge Questionnaire - Revised” (DKQ-R)^12^ and “Diabetes Knowledge Test”^13^. The majority of questions in this study’s final survey were drawn from the DKQ-R, as it was validated on a study population that was 42.6% Asian^12^. A license of permission to use the DKQ-R survey questions for this study was acquired on June 28, 2024.

Three of the 22 questions in the DKQ-R survey were replaced with questions from the Diabetes Knowledge Test by study investigators. Two were replaced because they were considered less relevant to the South Asian population, namely the questions regarding cooking with lard and the health quality of white bread. The third question regarding A1C levels was deemed redundant, as another question in the DKQ-R addressed similar content. The three questions modified for this study from the Diabetes Knowledge Test covered peripheral neuropathy symptoms, blood sugar monitoring at home, and the relationship between exercise and T2DM. These questions were chosen by investigators as they covered important aspects of T2DM that the DKQ-R did not include. The finalized survey was not independently revalidated due to the preliminary nature of this study.

The 22 diabetes knowledge assessment questions in the disseminated survey were “yes or no” format and were later grouped by study investigators into three categories—diabetes general knowledge (DK), diabetes management (DM), and complication management (CM)—for analysis. DK questions assessed basic understanding of the disease. DM questions evaluated participant knowledge about symptoms of T2DM, how lifestyle factors impact the diagnosis, and early signs of disease progression. CM questions elicited understanding about the impact of uncontrolled T2DM on various other organ systems. There were eight DK, eight DM, and six CM questions in the final survey.

The primary outcome of interest in this study was accuracy of responses to the 22 diabetes knowledge assessment questions, which were stratified into three predefined categories: DK, DM, and CM. Predictor variables included sex, level of education, relationship to the healthcare system, family history of T2DM, and self-reported clinical diagnoses, which were analyzed to assess their associations with DK, DM, and CM scores. Other demographic variables including age and ethnicity were collected for descriptive purposes but were not included in the primary analyses. 

### Educational brochure

Once surveys were completed, participants had the option to review their responses and view explanations for the correct answers to each question. Additionally, there was a link to an educational brochure created by the investigators, that summarized the definition, diagnosis, complications, treatment, and prevention of T2DM. The survey question explanations and educational brochure were meant as optional learning tools for participants post survey completion, and had no bearing on actual survey results.

### Statistical analysis

All statistical analyses were conducted using the SAS Software for Windows Version 9.4 (Cary North Carolina, USA). Descriptive statistics were presented as means and standard deviations for continuous variables, along with frequencies and proportions for categorical variables. Independent t-tests were conducted to assess the difference in outcomes between binary factors. Analysis of variance (ANOVA) was conducted to assess the difference in outcomes among nominal factors (e.g., educational levels). When the overall ANOVA test was significant, post-hoc analyses were conducted to identify significant pairwise comparisons. Effect sizes for group comparisons were calculated using Cohen’s d to aid interpretation of the magnitude of differences. Multivariable general linear model analyses were conducted to assess the joint effect of variables on the three question categories—DK, DM, and CM. Two-way interactions were assessed for statistical significance. Interactions among variables were also considered. These interactions include highest educational level and diagnosis of T2DM and highest educational level and working in a healthcare setting. The interaction term was retained in the final model only if it achieved statistical significance. All statistical tests were two-sided. A p-value of < 0.05 was considered to be statistically significant.

## Results

### Participant demographics

Among the 219 respondents, four were not U.S. citizens or permanent residents and were removed from the final analysis. As a result, 215 respondents were utilized for data analysis. There was no missing data in our dataset as participants were unable to submit the Google Forms survey if they did not answer all questions. Survey response rate could not be calculated due to the snowball sampling strategy. Table 1 summarizes demographic characteristics of the survey respondents. More than half of the respondents (70.2%) were females. The average age of respondents was 46.13 (standard deviation = 13.72) years and 51.6% completed graduate education. The majority of respondents had ethnic backgrounds from India (96.7%). 17.2% of participants had a diagnosis of T2DM, while 27.9% were told that they were at risk for developing this condition by a healthcare provider. 76.3% of participants had a parent, grandparent, or sibling diagnosed with T2DM and 24.2% of participants worked in a healthcare setting.

**Table 1:** Sociodemographic and Clinical Characteristics of Participants (*n* = 215).

Characteristic	*n*	%
Age, years	46.13 ± 13.72	N/A
Sex		
Female	151	70.2
Male	64	29.8
Ethnicity		
Indian	208	96.7
Pakistani	5	2.3
Bangladeshi	1	0.5
Sri Lankan	1	0.5
Highest level of education		
Less than high school	0	0
High school graduate	6	2.8
Associate degree	2	0.9
Bachelor’s degree	96	44.7
Graduate education or above	111	51.6
Working in healthcare setting	52	24.2
Healthcare access		
Annual physical exam within past year	184	85.6
Clinical Factors		
Diagnosed with T2DM	37	17.2
Told at risk for T2DM by provider	60	27.9
Family hx of T2DM	164	76.3

### Overall participant knowledge survey responses

Of the 22 knowledge questions in the survey, 17 questions were answered with an accuracy rate greater than 80%. Table 2 presents the correct response percentage for each of the survey questions. Males and females answered DK, DM, and CM questions with no statistically significant difference in regards to the percentage of correct responses. Similarly, there was no statistically significant difference in survey responses among those with a high school or associate’s degree, bachelor’s degree, or graduate degree.

**Table 2:** Survey Questions Answer Key with Percentage of Correct Responses (*n* = 215).

Question	Answer Key	Correct Response (%)
Diabetes Knowledge (DK)		
The body needs insulin to use the sugar that a person eats.	Yes	90.2
When someone has diabetes, their children are more likely to get diabetes.	Yes	90.2
A fasting blood glucose level of 100 is too low.	No	88.8
Shaking and sweating are signs of high blood glucose.	No	45.1
Frequent urination and thirst are signs of low blood glucose.	No	57.2
A1C measures a 3-month average of blood glucose levels.	Yes	90.2
After eating lunch, a glucose level of 200 is too low.	No	94.0
Blood testing is the best method for home glucose testing.	Yes	89.8
Diabetes Management (DM)		
It is safe for people with diabetes to wear flip flops.	No	55.8
A person with diabetes can eat as much as they want if it is sugar-free.	No	96.7
Stress lowers blood glucose levels.	No	87.4
Smoking will raise the chances of diabetes complications.	Yes	94.9
People with diabetes should increase the amount of trans-fat in their diets.	No	85.6
Numbness and tingling may be symptoms of nerve disease.	Yes	90.2
Feeling angry can raise blood glucose levels.	Yes	54.9
Exercise can help control blood sugar levels.	Yes	99.1
Complication Management (CM)		
People with high glucose have problems with blood circulation in their legs.	Yes	91.6
People with high glucose can have damage to the kidneys.	Yes	94.0
Vaccines are highly recommended for people with diabetes.	Yes	75.4
People with diabetes should see the dentist twice a year.	Yes	80.9
Someone with diabetes is more likely to get heart disease.	Yes	91.2
People with diabetes should have their eyes checked every year.	Yes	96.3

### Diabetes knowledge (DK) questions

The DK questions were answered with the lowest accuracy on average, 80.7%, among all three categories of questions. More specifically, two of the most frequently missed questions in the survey came from this category. Only 45.1% of participants accurately identified “shaking and sweating are signs of high blood glucose” as a false statement. Additionally, only 57.2% of participants recognized “frequent urination and thirst are signs of low blood glucose” as an inaccurate statement. Participants with a significantly higher DK score were: those who had a diagnosis of T2DM (*p* = 0.0225, Cohen’s d = 0.42), those employed in healthcare settings (*p* = 0.0002, Cohen’s d = 0.60), and those at risk for T2DM (*p* = 0.0161, Cohen’s d = 0.40). However, there was no statistically significant difference in average DK score between those who had a family member diagnosed with T2DM and those who did not (*p* = 0.2306, Cohen’s d = 0.19).

### Diabetes management (DM) questions

The second analysis compares demographic factors associated with DM score. On average, the eight DM questions were answered with an 83.1% accuracy, the second lowest average among the three categories. The other two most commonly missed questions in the survey came from this category. Only 55.8% of people answered “false” for the statement: “it is safe for people with diabetes to wear flip flops.” Additionally, only 54.9% of participants identified “feeling angry can raise blood glucose levels” as a true statement. Overall, no statistically significant difference was found in the DM score in the following group comparisons: healthcare workers vs. non-healthcare workers, those with T2DM vs. those without the disease, those with family members with the condition vs. those without, those at risk for T2DM vs. those not at risk, and those with T2DM who had an annual exam in the past year vs. those without the condition who had an annual exam.

### Complication management (CM) questions

The highest accuracy of all question groups came from the CM category, which had an average of 88.2% accuracy for the 6 questions in this category. Those with T2DM (*p* = 0.0138, Cohen’s d = 0.45), those employed in healthcare settings (*p* = 0.0035, Cohen’s d = 0.47), and those with a family member with T2DM (*p* = 0.0123, Cohen’s d = 0.48) demonstrated a statistically higher CM score compared to their counterparts.

### Multivariable analysis of variables for DK, DM, and CM categories

Multivariable general linear model analyses were conducted to assess the joint effect of covariates on the outcomes. The covariates included highest educational level achieved, employment in the healthcare setting, diagnosis of T2DM, and sex. There is a statistically significant association between family history of T2DM and whether the respondent has a diagnosis of T2DM (odds ratio 14.8, 95% confidence interval: 1.98, 111.42, *p* = 0.0007). There is also a statistically significant association between risk for T2DM and whether the respondent has a diagnosis of T2DM (odds ratio 10.8, 95% confidence interval: 3.98, 21.14, *p* < 0.0001). Therefore, the family history of T2DM and the at risk for T2DM covariates were not included in the multivariable model. All two-way interaction terms were not statistically significant and were removed from the model as well.

Table 3 presents the multivariable analysis results and demonstrates that respondents with a higher DK score were those who work in healthcare settings (*p* < 0.0001) and those who have a diagnosis of T2DM (*p* = 0.0090). A similar pattern was observed in the DM score where those who work in healthcare settings (*p* = 0.0012) and those who have a diagnosis of T2DM (*p* = 0.0030) performed better on the DM questions. None of the covariates, including highest educational level achieved, employment in the healthcare setting, diagnosis of T2DM, and sex, were significant predictors for the CM management questions.

**Table 3:** Multivariable Analysis of Variables for the Three Scores.

Variable	Diabetes Knowledge Score		Diabetes Management Score		Complication Management Score	
	β (SE)	*p*-value	β (SE)	*p*-value	β (SE)	*p*-value
Educational level						
HS/Associate (Reference)						
Bachelor’s degree	0.20 (0.39)	0.6070	0.01 (0.39)	0.9990	−0.15 (0.38)	0.6790
Graduate or above	0.54 (0.39)	0.1690	0.24 (0.39)	0.5340	0.05 (0.38)	0.8990
Work in a healthcare setting						
Yes (Reference)						
No	−0.80 (0.18)	< 0.0001	−0.58 (0.17)	0.0012	−0.19 (0.17)	0.2570
Sex						
Male (Reference)						
Female	−0.01 (0.17)	0.9590	0.26 (0.17)	0.1290	0.27 (0.16)	0.1040
Diagnosed with T2DM						
Yes (Reference)						
No	−0.50 (0.19)	0.0090	−0.58 (0.19)	0.0030	−0.24 (0.19)	0.1930

*β coefficients represent adjusted differences in scores derived from multivariable general linear models controlling for educational level, employment in a healthcare setting, sex, and diabetes diagnosis status. Reference categories are indicated in parentheses. SE=standard error.

## Discussion

This preliminary cross-sectional study indicates that our sampled US South Asian population demonstrated an appreciable understanding of T2DM pathology, management, and complications as most survey questions were answered with a greater than 80% accuracy.

Both univariate and multivariate analyses were conducted to assess how participants’ demographic characteristics affected DK, DM, and CM scores. In the multivariate analysis, education, healthcare employment, T2DM status, and sex were included, while family history and at-risk status were excluded due to their strong association with T2DM diagnosis. For this reason, multivariate analysis was not conducted to confirm the association found in the univariate analysis between increased DK scores and those at risk for T2DM as well as increased CM scores and those with a family history of T2DM. Furthermore, multivariate analysis was concordant with univariate analysis findings that sex and education level do not impact scores in any one of the three question categories.

For the DK questions, the univariate analysis reported significant scores in those who work in healthcare settings and those with a T2DM diagnosis. These associations remained significant in the multivariate analysis, indicating that these factors independently predict better general knowledge of T2DM.

Univariate analysis of the DM category revealed no statistically significant differences across demographic groups. In the multivariate analysis, however, healthcare employment (*p* = 0.0012) and diagnosis of T2DM (*p* = 0.0030) were independent predictors of higher DM scores, suggesting that these associations were masked previously. Based on these results, those working in healthcare and those with a diagnosis of T2DM have a better understanding of T2DM lifestyle factors and early signs of T2DM disease progression.

Lastly, for the CM questions, the univariate findings revealed significant scores in those who had a diagnosis of T2DM, and those employed in healthcare settings. However, these associations were no longer significant in the multivariate analysis. These results demonstrate that the initial associations identified between higher CM question score and healthcare employment and T2DM diagnosis respectively, were driven by the other demographic variables. Ultimately, it cannot be concluded that working in the healthcare field or having a diagnosis of T2DM offer patients an improved understanding of T2DM complications.

Additionally, the two lowest scoring questions in this survey pertained to hypoglycemia, a serious consequence of T2DM treatment^14^. Only 45.1% of participants correctly identified “shaking and sweating are signs of high blood glucose” as false, and just 57.2% recognized “frequent urination and thirst are signs of low blood glucose” as inaccurate. Misconceptions remain that those with T2DM do not experience hypoglycemic episodes^14^. However, there is evidence that the prevalence of hypoglycemia in insulin-treated T2DM patients may equal to or exceed those in type 1 diabetes patients^14^. Considering the serious cardiovascular and cognitive risks associated with hypoglycemic episodes including arrhythmias, dementia, and myocardial ischemia, the lack of knowledge about this symptom among our study participants is concerning^14,15^. It is important that providers discuss hypoglycemic symptoms with T2DM patients and also recommend blood sugar monitoring strategies and interventions for hypoglycemia reversal. Prior studies demonstrate that one to two weeks of continuous glucose monitoring in patients with T2DM, can identify more hypoglycemic episodes than symptom assessment alone, providing useful data for clinicians to guide medication adjustments^15^. Additionally, patients with T2DM on insulin should be educated on immediate management of hypoglycemia, including the use of glucose-containing foods or glucagon when appropriate^15^.

In comparing our findings with studies conducted in other countries, one U.K. cohort study demonstrated that their participants were found to be well-educated about the impact that certain foods had on the overall management of their diabetes^16^. Similarly, the participants of this study also seemed to perform well on questions pertaining to the effect of diet on T2DM. Namely, the questions “A person with diabetes can eat as much as they want if it is sugar-free,” and “people with diabetes should increase the amount of trans-fat in their diets,” were answered with an accuracy rate of greater than 85%. By contrast, while this study demonstrated an overall strong understanding of T2DM with the given participant population of South Asians in the U.S., a population study in India found that only 43.2% of their participants had heard about diabetes^17^. Together, these findings suggests that while the South Asian diaspora in the US may demonstrate stronger baseline awareness of T2DM, diabetes knowledge remains uneven across geographic and population groups.

Given these differences in baseline awareness, recent literature suggests that improving diabetes knowledge and management may require approaches beyond traditional clinic-based education, particularly in underserved communities. One example is the SEVAK Project, which used locally trained community members to provide health education, screening, and prevention-oriented support for diabetes and related conditions^18^. This model shows how community-based workers may help extend health education and early detection into settings with limited access to formal healthcare^18^. Other studies have focused on educational models that use nontraditional instructors. In Guyana, high school students were trained to participate in community health education and chronic disease monitoring, suggesting that youth can play a meaningful role in promoting disease awareness at the local level^19^. Similarly, a structured health promotion curriculum implemented among Indian youth improved non-communicable disease health literacy compared with controls^20^. Together, these studies suggest that decentralized, community-centered educational strategies may be useful for increasing diabetes awareness and supporting prevention efforts in underserved populations.

Future research should examine how South Asians living in the United States engage in lifestyle behaviors that influence diabetes control, particularly physical activity and diet. While novel community-based educational strategies in foreign countries may improve diabetes knowledge, additional work is needed to determine whether these approaches translate into sustained behavior change in the US South Asian population. Prior studies suggest that South Asian immigrants may be less likely to meet recommended physical activity levels compared with other ethnic groups and may consume diets that reflect a combination of traditional and Western eating patterns high in refined carbohydrates, saturated fats, and sugar-sweetened beverages^21–24^. These findings suggest that future interventions should extend beyond improving T2DM knowledge alone, as high factual awareness does not necessarily translate into improved health outcomes if patients continue unhealthy behaviors. Ideal interventions would incorporate culturally tailored, community-centered strategies that support lifestyle modification, such as nutrition counseling, physical activity monitoring, and longitudinal behavioral support.

### Limitations

The primary limitation of this study is the use of convenience sampling, as the survey was initially distributed to friends and family of study investigators. However, since the study was voluntary, anonymous, and disseminated by initial participants to other contacts they knew, the final study sample included participants completely unknown and unrelated to investigators. This recruitment strategy introduces selection bias and limits the generalizability of the findings. The US census bureau does not provide education statistics specifically for combined South Asian populations nor does it provide detailed estimates for graduate-level education. Instead, available data is limited to a select few South Asian racial subcategories and report educational attainment as bachelor’s degree or higher^25,26^. As there is no specific delineation of all South Asian populations, it is difficult to determine whether the educational attainment observed in our sample accurately reflects the broader South Asian population in the US. It is possible that this study’s recruitment strategy resulted in a subgroup of South Asians who are more highly educated, English-speaking, and connected to healthcare networks than the broader South Asian population in the United States. This leads to the observed high proportions of graduate degrees in the study sample. These characteristics may contribute to the higher DK, DM, and CM baseline knowledge. As a result, this may overestimate the awareness level of T2DM when compared to the broader South Asian population in the US. Nevertheless, the current study still provides useful insights into T2DM related knowledge among a highly educated South Asian subgroup in the US.

Additionally, while the study sample was meant to be representative of South Asian people living in the United States, people of Indian ethnicity were slightly overrepresented in this study. 96.7% of study respondents were of Indian origin, while prior studies estimate that Indian Americans make up about 80% of the South Asian population^27^— a discrepancy to consider when generalizing these study results to all South Asians in the United States. Future research efforts should emphasize recruiting participants from other South Asian backgrounds to establish a more balanced representation of the US South Asian population.

Furthermore, there is potential for recall bias in this study as investigators did not verify participants’ responses to demographic questions. In particular, the questions “Have you had an annual physical examination within the past year?” and “Have you ever been told you are at risk for diabetes by your healthcare provider?” could be particularly susceptible to issues of recall.

Lastly, the survey disseminated to participants included questions selected from two previously validated surveys. Since the finalized survey used in this preliminary study was not independently revalidated, there are limitations to the internal consistency and reliability of the survey results. Future goals include revalidating the survey used in this study to allow for more robust conclusions from the data.

## Conclusion

Diabetes education is often viewed as an effective strategy for improving disease outcomes in people with T2DM, a condition that disproportionately impacts those of South Asian origin. The preliminary study findings suggest that the sampled South Asian community had a relatively strong understanding of T2DM and its management, except for the complication of hypoglycemia. In particular, healthcare workers and those with a diagnosis of T2DM demonstrated a better general understanding of T2DM and how lifestyle factors influence the disease, after adjusting for other variables. The participants in this study were highly educated and predominantly of Indian origin, making the results less generalizable to the broader South Asian population which is more socioeconomically and ethnically diverse^25–27^. Future education efforts should focus on how to recognize and manage hypoglycemic symptoms as this complication can be fatal. Furthermore, it is important to explore community-centered intervention strategies targeting physical activity and diet, as these behaviors are often suboptimal in the U.S. South Asian population. Identifying and implementing culturally tailored interventions for South Asians in the United States living with T2DM remains a critical priority to improve long-term health outcomes.

## Data Availability

The data that support the findings of this study are available from the corresponding author upon reasonable request.
